# Polygenic risk score analysis suggests hypothyroidism as a risk factor for Alzheimer’s disease

**DOI:** 10.1002/alz.088329

**Published:** 2025-01-03

**Authors:** Ignazio Piras, Marcus Naymik, Janith Don, Nicholas J. Schork, Don Saner, Matthew J. Huentelman

**Affiliations:** ^1^ The Translational Genomics Research Institute (TGen‐ an Affiliate of City of Hope), Phoenix, AZ USA; ^2^ Banner Alzheimer’s Institute, Phoenix, AZ USA

## Abstract

**Background:**

Alzheimer’s Disease (AD) is characterized by cognitive decline due to synaptic loss and neuron death, with amyloid‐β plaques and neurofibrillary tau tangles as key pathological hallmarks. Although genetics account for about 70% of AD risk, modifiable factors also significantly contribute to AD and dementia onset and AD‐resilience. Utilizing the All of Us (AoU) cohort, this study explores the relationship between clinical conditions, quantitative phenotypes (including lab tests and anthropometric measurements), and AD risk, shedding light on potential preventative measures.

**Method:**

We selected a subset of non‐AD affected individuals from the AoU Controlled Tier Dataset v7 for analyzing medical conditions and measurements related to AD. Polygenic Risk Scores (PRS) were calculated to assess AD risk, which were then associated with medical conditions and measurements using linear regression models and accounting for biological sex and age. A replication study was conducted in the UK Biobank to validate the findings, particularly focusing on the association between AD‐PRS and hypothyroidism prevalence.

**Result:**

We found significant association between AD‐PRS and nine conditions, with the most significant hypothyroidism and acquired hypothyroidism (adj‐p < 1.3^‐06^). The AD‐PRS was significantly lower in participants with these conditions. Additionally, an investigation into 355 measurement variables revealed a significant association for eighteen variables, with height and blood pressure showing a positive correlation with AD‐PRS. Thyrotropin level (TSH) exhibited a negative correlation, aligning with hypothyroidism’s association with lower PRS. This association remained significant even after accounting for hypothyroidism medications in the analysis, and was further confirmed in a replication study using the UK Biobank data (n = 390,543; p = 1.3^‐23^)

**Conclusion:**

Our results seem to demonstrate that the absence of HPT might be a factor enhancing AD resilience since it can balance the genetic risk factors, as non‐affected individuals exhibited a significantly higher AD‐PRS than HPT‐affected individuals.

